# Isolation and Identification of Lactic Acid Bacteria from Cow Milk and Milk Products

**DOI:** 10.1155/2021/4697445

**Published:** 2021-08-10

**Authors:** Yeshambel Taye, Tadesse Degu, Haben Fesseha, Mesfin Mathewos

**Affiliations:** ^1^Raya Kobo Woreda Animal Health Disease Surveillance and Control Expert, Kobo, Ethiopia; ^2^Raya Kobo Woreda Animal Health Expert, Kobo, Ethiopia; ^3^School of Veterinary Medicine, Wolaita Sodo University, P.O Box: 138, Wolaita Sodo, Ethiopia

## Abstract

Lactic acid bacteria (LAB) have long been consumed by people in several fermented foods such as dairy products. A study was conducted on lactating dairy cows to isolate and characterize LAB from dairy products found in and around Bahir-Dar city, North Western Ethiopia. Milk and milk products were randomly collected from dairy farms, milk vending shops, individual households, and supermarkets for bacteriological investigations. A total of sixteen samples were taken from different sources and cultured on different selective media: de Man, Rogosa, and Sharpe (MRS) agar for *Lactobacillus* spp.; M17 agar for *Lactococcus* spp.; Rogasa SL agar for *Streptococci* spp.; and MRS supplemented with cysteine (0.5%) for *Bifidobacteria* spp. Different laboratory techniques were implemented for LAB isolation and identification. A total of 41 bacterial isolates were grouped under five different genera of LAB and *Bifidobacteria* spp. were identified based on the growth morphology on the selective media, growth at a different temperature, gas production from glucose, carbohydrate fermentation, and other biochemical tests. LAB genera such as *Lactobacillus*, *Lactococcus*, *Leuconostoc*, *Pediococcus*, *Streptococcus*, and *Bifidobacterium* spp. were isolated and identified from raw milk, cheese, and yogurt. Based on the current study, the majority of the LAB (24.38%) was isolated from cheese and yogurt. Among these, *Lactobacillus*, *Lactococcus* (21.94%), *Streptococcus* (19.51%), *Leuconostoc* (14.64%), *Bifidobacteria* (12.19%), and *Pediococcus* (7.31%) spp. were also identified from these products. Furthermore, based on the bacterial load count and different identification methodologies, our study revealed that *Lactobacillus* spp. were the dominant LAB isolated from milk and milk products. As a result, since there are few studies on the isolation and identification of lactic acid bacteria from dairy products in Ethiopia, more research studies are needed to complete the identification and characterization to species level and their possible role as probiotics.

## 1. Introduction

People have historically consumed lactic acid bacteria (LAB) in fermented foods such as dairy products. LAB are currently the subject of extensive international research due to their critical involvement in most fermented foods, as well as their potential to create antimicrobial chemicals that promote probiotic characteristics [1] including antitumor activity [2], alleviation of lactose intolerance [3], a decrease of serum cholesterol [4], gut microflora stabilization [5], and stimulation of the immune system [6]. In the production of fermented milk, the LAB strain produced for exopolysaccharides is used to improve their viscosity and smoothness [7]. Some LAB strains are known to produce the presumed effects of mannitol which promote health [8].

Probiotics are live bacteria or yeasts that, when given in sufficient proportions, provide a health benefit to the host [9, 10]. To date, with the growing interest in health consciousness, the concept of probiotic foods has gotten a lot of attention. A large number of probiotic species and strains belong to the genera *Lactobacillus* and *Bifidobacterium*. Other groups of the LAB (*Streptococcus* and *Enterococcus*), *Bacillus, Propionibacterium*, and the yeast *Saccharomyces* are being used as probiotic microorganisms [11, 12].

The gastrointestinal tract (GIT) consists of diverse microorganisms mainly dominated by bacteria. Other archaeal domains, yeasts, and molds are also found in the GIT [13]. Probiotic microorganisms are thought to have a wide range of health benefits. They exert antimicrobial activity against enteric pathogenic bacteria; stimulate mucosal and systemic immune responses of the host; provide anticolon cancer, anticholesterol, and anti-inflammatory effects; and improve nutritional status. They also reduce symptoms of diarrhea, lactose intolerance, and allergic reactions [13]. So, multistrain or multispecies probiotic mixes are becoming more popular because they can give additive or even synergistic effects, as well as a broader spectrum of health advantages [14, 15].

The benefits of probiotic intervention in the gut microbiota in maintaining and restoring health are becoming more well recognized, drawing increased scientific interest [16–18]. The expected probiotic strains should be assessed for possession of physiological and genetic traits and technological properties beneficial to the host. Probiotic organisms must be able to live in the presence of gastric acid and bile, as well as adhere to and colonize the gastrointestinal epithelial layer. They also should be safe and nonpathogenic to the host [19].

Probiotics are the key components of functional meals that account for over 65 percent of the global functional food market. LAB, such as *Lactobacillus*, *Bifidobacterium*, and *Enterococcus* spp., are among the live bacteria included in probiotic supplements. Within the phylum Firmicutes, LAB are made up of various genera. LAB includes the genera of *Carnobacterium, Enterococcus, Lactobacillus, Lactococcus, Lactosphaera, Melissococcus, Oenococcus, Pediococcus, Streptococcus, Tetragenococcus, Vagococcus,* and *Weissella* [20–24].

Gram-positive lactic acid-producing bacteria that belong to the phylum Actinobacteria are *Aerococcus, Microbacterium,* and *Propionibacterium* [25] as well as *Bifidobacterium* [21, 26]. Gram-positive bacteria that ferment carbohydrates into energy and lactic acid are members of the LAB family [22, 27]. Depending on the organism, the metabolic processes are different if glucose is the major source of carbon: homofermentative bacteria, including *Lactococcus* and *Streptococcus* spp., give two lactates from a single glucose molecule, whereas glucose molecules are transformed into lactates with ethanol and carbon dioxide (i.e., *Leuconostoc* and *Weissella* spp.) [22, 28, 29].

LAB also produces tiny organic molecules that give the fermented product its aroma and flavor [28]. The study on isolation and characterization of LAB or probiotics was so far not well studied in milk and milk products in Ethiopia. Considering the importance of probiotic bacteria in milk and milk products, only a few works have been done on probiotics in the study area. Therefore, LAB from cow milk and milk products were isolated and identified in this research.

## 2. Materials and Methods

### 2.1. Study Animals and Design

Lactating dairy cows were the study animals that were managed in a semi-intensive way. A cross-sectional study was conducted from November 2015 to March 2016 to isolate and characterize LAB from milk and milk products gathered from lactating dairy cows located at different farms of Bahir Dar district.

### 2.2. Study Methodology

#### 2.2.1. Procedures for Collecting and Handling Samples

For the bacteriological analysis, a total of 16 milk and milk products were collected (7 raw milk samples from dairy farms (3), households (2), and milk samples from vending shops (2); 4 cheese samples from households and supermarkets; and 5 yogurts from households, milk vending shops, and cafeterias). Early in the morning, a total of 300–500 ml of milk and milk products were collected from dairy farms, individual households, supermarkets, milk vending shops, and cafeterias (farmers) by using a sterile glass bottle. Then, the samples were labeled correctly, stored at +4^o^C, and the samples were cultivated bacteriologically.

#### 2.2.2. Isolation and Identification of LAB

Bacterial isolation was performed by preparing serial dilutions of the samples with sterilized maximum recovery diluents (pH = 7), 0.1 ml of the dilution was spread on de Man, Rogosa, and Sharpe (MRS) agar for LAB isolation, M17 broth + agar powder for *Lactococcus* isolation (HiMedia, Mumbai, India), Rogasa SL agar for *Streptococcus thermophilus*, and MRS agar + cysteine (0.5%) for *Bifidobacterium*. The agar plates were incubated at 30°C and 37°C under aerobic and anaerobic conditions for 48–72 h [30].

Anaerobic jars were used to achieve the anaerobic condition. Viable aerobic and anaerobic counts of the dairy products were determined. The isolation was obtained by morphological characteristics (colony and cell morphology), on the selective media, and biochemical tests used were Gram reaction, catalase test, production of acid from glucose, and growth at different temperatures. Only Gram-positive bacteria with catalase-negative responses were found, and representative isolates were purified by streaking them over the same agar substrate several times. Growth at various temperatures, hetero-, and homofermentative activity (using MRS broth) with inverted Durham tubes in MRS broth was determined for the Gram-positive, catalase-negative rods. Following that, isolates from dairy product samples were selected for further identification based on the previous assays by Harrigan and MacCance [31]. Moreover, all isolates were preserved in brain heart infusion (BHI) with 15% glycerol to detect the carbohydrate fermentation profile of the isolates (Supplementary Materials).

At the end of incubation (37°C for 48 h), the LAB were selected based on their morphology in their selective media such as colony character and biochemical profiles. Moreover, the carbohydrate fermentation profiles of isolates were determined with the micromethod according to Roy and Ward [32]. The details for isolation and identification of the LAB are included in a separate supplementary file.

#### 2.2.3. Enumeration of LAB

Different selective mediums were used to culture the LAB such as MRS, M17, and MRS supplemented with cysteine for *Lactobacillus*, *Lactococcus*, and *Bifidobacteria*, respectively, described by Oxoid Manual [33]. For *Bifidobacteria* spp., the colony count methodology was used to enumerate presumptive LAB at 30°C and 37°C under both aerobic and anaerobic conditions. The technique of counting was as follows. First, tenfold serial dilution of 9 ml maximum recovery diluents to 1 ml of the sample was done up to 10^−8^, and accordingly, all of the serially diluted samples were cultured on the MRS agar, M17 agar, Rogasa SL agar, and MRS + 0.5% cysteine for the cultivation of all *Lactobacillus*, *Lactococcus*, *Lactobacillus, Streptococcus thermophilus,* and *Bifidobacteria* spp., respectively. Then, incubate at 37°C for 48 h, and 10^−6^ dilutions were selected for colony characterization and counted accordingly. During the test, representative colonies were stored in glycerol culture at 20^o^C and kept for further investigation.

### 2.3. Data Analysis

Data from the laboratory were stored and transferred into Microsoft Excel 2016 Spreadsheet analyzed using STATA version 13. The outputs (bacterial isolates) were presented using frequency and percentage.

## 3. Results

### 3.1. LAB Isolation

**Figure 1:**
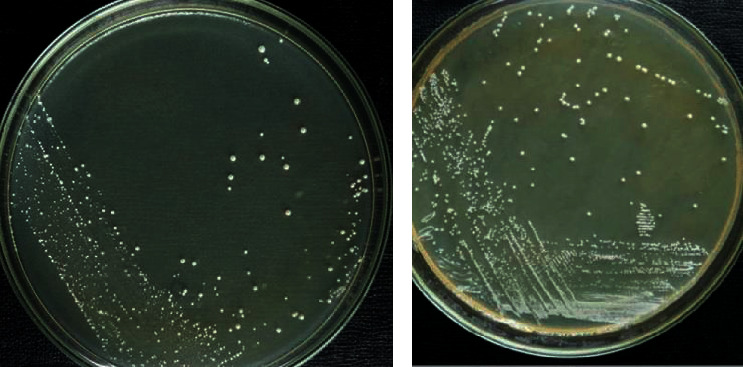
(a) Typical isolated colonies of *Lactobacillus* spp. on MRS media. (b) *Lactococcus* spp. on M17 media.

According to this study, identification of the LAB was performed through morphological characteristics. Besides, other biochemical tests and all of the LAB were identified to its generic level in that *Lactobacillus* spp. were found as whitish, small to large size, the circular margin on MRS media (Figure 1(a)), and *Lactococcus* spp. were found to be creamy white to yellowish color, small to large in size, and circular margin on M17 media (Figure 1(b)).

Also, Gram stain characteristics of the bacterium revealed that all of the bacteria were Gram-positive, cocci shaped, and have different arrangements: *Streptococcus* spp. were found in chain arrangement; *Leuconostoc* spp. were found more in paired arrangement; *Pediococcus* observed were having tetrads and *Bifidobacteria* spp. were observed as pleomorphic filamentous-like flagella which were observed under oil immersion. Besides, *Bifidobacteria* spp. were identified based on the growth morphology on the selective media, growth at a different temperature, gas production from glucose, and carbohydrate fermentation. Our result revealed that a total of 41 bacterial genera were isolated and categorized into five different genera of LAB (Table 1).

Moreover, 5 (12.19%) of the bacteria isolated from the raw cow milk were *Lactobacillus* spp., followed by *Lactococcus* and *Streptococcus* spp., but *Leuconostoc* spp. were found in low percentage. However, in cheese, *Lactococcus* spp. were found in a higher proportion than the other bacteria, and in the case of yogurt, *Lactobacillus* spp. were found in a higher proportion (Table 2).

### 3.2. Enumeration of LAB from Milk and Milk Products

According to the enumeration of LAB, bacterial load was counted from different milk and milk products. Thus, *Lactobacillus* spp. were found to be high (2.3 × 10^8^ CFU/ml) in yogurt and low (4.5 × 10^7^ CFU/ml) in raw milk; similarly, a higher load of *Lactococcus* and *Bifidobacteria* spp. was counted from yogurt (Table 3).

## 4. Discussion

Based on the current study, the majority (24.38%) of the LAB isolated from the different milk and milk products was containing *Lactobacillus*, *Lactococcus* (21.94%), *Streptococcus* (19.51%), *Leuconostoc* (14.64%), *Bifidobacteria* (12.19%), and *Pediococcus* (7.31%) spp. Savadogo et al. [34] and Harun-ur-Rashid et al. [35] also found six genera of LAB from traditional fermented milk, including *Leuconostoc, Lactococcus, Lactobacillus, Enterococcus, Streptococcus,* and *Pediococcus* spp. Similarly, Abd El Gawad et al. [36] have also identified five different genera of LAB : *Lactobacillus* (30%), *Leuconostoc* (26%), *Enterococci* (20%), *Streptococcus* (18%), and *Aerococcus* (18%) spp. Also, a comparable figure was reported by Abdullah and Osman, [37] where the genus *Lactobacillus* was dominant in all samples tested (69.23%) followed by the genus *Lactococcus* (19.23%) and the genus *Pediococcus* (11.53%).

Moreover, this study has similar findings with El-Shafei et al. [38] and Abd El Gawad et al. [36] who identified a higher proportion of *Lactobacillus* spp. from milk products. *Lactobacillus* spp. can survive in a highly acidic environment with a pH of 4 to 5 or even lower, and it is *Lactobacillus* that is responsible for the final stages of fermentation in the goods as a result of these traits. This also demonstrated that *Lactobacillus* spp. survive in low pH environments [39].

Based on the current study of LAB enumeration from different milk and milk products, it revealed that *Lactobacillus* spp. were presumptively counted as 4.5 × 10^7^, 7.7 × 10^7^, and 2.3 × 10^8^ CFU/ml from raw cow's milk, cheese, and yogurt, respectively. However, a load of *Lactococcus* spp. was counted as 1.12 × 10^7^, 8.01 × 10^7^, and 2.75 × 10^9^ CFU/ml from raw cow's milk, cheese, and yogurt, respectively. In the same way, *Bifidobacteria* spp. were counted as 2.34 × 10^7^, 2.84 × 10^7^, and 1.72 × 10^8^ CFU/ml from the mentioned milk and milk products. These findings were in accordance with the earlier report of Khedid et al. [40] from Morocco, who reported that *Lactobacillus* spp. counting load were 7.5 × 10^6^ CFU/ml, from camel milk. However, a *Lactococcus* spp. counting load in the present study was higher than the report of Khedid et al. [40], which is 7.6 × 10^6^ CFU/ml.

Moreover, the finding of Savadogo et al. [34] revealed that LAB were found in large numbers in all samples from various governorates, ranging from 1.02 × 10^−2^ to 9.89 × 10^−2^ CFUg^−1^. All these populations rose from around 10^2^ CFUg^−1^ to 10^4^ CFUg^−1^. These findings might be due to the employment of primitive manufacturing procedures in the production of many traditional foods, with the primary risk factors being the use of contaminated raw materials that have not been pasteurized, as well as insufficient fermentation and storage conditions. Despite the fact that the LAB group dominated the microbiota, a final population of roughly 10^6^ to 10^7^ CFUg^−1^ was achieved in all samples.

The most frequent microbial group detected in milk was LAB, which is relevant given its role in fermentation and the generation of lactic acid and antibacterial compounds, as well as its potential use as a milk starter in standardized production. Gram-stain reaction, cell shape, cellular organization, generation of acid from glucose and lactose, production of gas from glucose, and catalase activity were used to isolate, identify, and classify LAB strains [36, 41].

El-Shafei et al. [38] also reported the preponderance of *Lactobacillus* spp. was higher among isolated LAB because milk and milk products constitute a varied mixture of diverse microorganisms. Furthermore, cow milk, which is widely used to make fermented milk, may promote the growth of these species. *Lactobacillus* spp. can survive in a highly acidic environment with a pH of 4 to 5 or even lower, and it is *Lactobacillus* spp. that is responsible for the final stages of fermentation in products because of these qualities. This also demonstrated that *Lactobacillus* spp. survive in low pH environments [39].

## 5. Conclusion

The current study revealed that most of the isolated LAB species were grouped into five genera including *Lactobacillus*, *Lactococcus*, *Leuconostoc*, *Pediococcus*, and *Streptococcus,* and *Bifidobacteria* spp. and *Lactobacillus* spp. were the most common LAB isolated from various milk and milk products among the identified genera. Moreover, *Lactobacillus* spp. were the commonly isolated bacterial species from raw cow milk and yogurt; however, *Lactococcus* and *Bifidobacteria* spp. were found in a higher proportion in cheese. Based on the enumeration of LAB, *Lactobacillus, Lactococcus,* and *Bifidobacteria* spp. were counted in higher proportion from yogurt and raw milk. Thus, further study on the identification and characterization of LAB to strain level should be done. The health and economic benefits of the identified LAB should be given more attention in order to improve community health. The possible application of these LAB as probiotics should be further tested on different species of experimental animals.

## Figures and Tables

**Table 1:** LAB isolated from various milk and milk products.

Genus	Raw milk	Cheese	Yoghurt	Total
*Lactobacillus* spp.	5	2	3	10
*Lactococcus* spp.	4	3	2	9
*Streptococcus* spp.	4	2	2	8
*Leuconostoc* spp.	2	2	2	6
*Pediococcus* spp.	3	0	0	3
*Bifidobacteria* spp.	3	1	1	5

**Table 2:** Proportions of LAB isolated from milk and milk products.

Sample	*Lactobacillus* spp.	*Lactococcus* spp.	*Streptococcus* spp.	*Leuconostoc* spp.	*Pediococcus* spp.	*Bifidobacteria* spp.	Total
Raw milk	5 (12.19)	4 (9.75%)	4 (9.75%)	2 (4.88%)	3 (7.3%)	3 (7.31%)	21 (51%)
Cheese	2 (4.88)	3 (7.3%)	2 (4.88%)	2 (4.88%)	0 (0%)	1 (2.44%)	10 (24.38%)
Yoghurt	3 (7.31%)	2 (4.88%)	2 (4.88%)	2 (4.88%)	0 (0%)	1 (2.44%)	10 (24.38%)
Total	10 (24.38%)	9 (21.94%)	8 (19.51%)	6 (14.64%)	3 (7.31%)	5 (12.19%)	41

**Table 3:** Bacterial load in different milk and milk products.

Genus of bacteria	Media	Df	Colony-forming unit (CFU/ml)		
			Raw milk	Cheese	Yoghurt
*Lactobacillus*	MRS	10^6^	4.5 × 10^7^	7.7 × 10^7^	2.3 × 10^8^
*Lactococci*	M17	10^6^	1.12 × 10^7^	8.01 × 10^7^	2.75 × 10^9^
*Bifidobacteria*	MRS + cys	10^6^	2.34 × 10^7^	2.84 × 10^7^	1.72 × 10^7^

Df = dilution factor.

## Data Availability

All the datasets generated or analyzed during this study are included in this manuscript.

## Abbreviations

ESP: Exopolysaccharide
FAO: Food and Agricultural Organization
GIT: Gastrointestinal tract
LAB: Lactic acid bacteria
MRS: De Man, Rogasa, and Sharpe
WHO: World Health Organization.

## Supplementary Materials

Supplementary Materials Procedure used to isolate, identify, and primary and secondary biochemical test to identify the lactic acid bacteria.
