# Supplementary material for: Isolation and Identification of Lactic Acid Bacteria from Cow Milk and Milk Products
Source: ScientificWorldJournal. 2021 Aug 10;2021:4697445. doi: 10.1155/2021/4697445 (PMC8371643; doi:10.1155/2021/4697445)
Supplement: Supplementary Materials — Procedure used to isolate, identify, and primary and secondary biochemical test to identify the lactic acid bacteria. [file 4697445.f1.pdf]

Supplementary file:

Procedure used to isolate, Identify and primary and secondary biochemical test used to identify the lactic acid bacteria

Table 1: Growth characteristics of lactic acid bacteria on selective Media and biochemical test

**Tests**	***Lactobacilli***	***Lactococci***	***Streptococci***	***Leuconostoc***	***Pediococcus***	***Bifidobacteria***
MRS	+	+	+	+	+	+
MRS+cysteine	+	+	+	+	+	+
M17	+	+	+	+	+	+
SL-Rogassa	+	+	+	+	+	+
Gram stain	+	+	+	+	+	+
properties	Rod<br>Chains	Cocci<br>In pairs and short chains	Cocci<br>Long chains	Cocci<br>Pairs	Cocci<br>Pairs or tetrads	Rods<br>Pleomorphic, filamentous
Catalase	-	-	-	-	-	-
Gas production from glucose	-	-	-	+	-	-
Incub. 20	-	+	-	+	+/_	-
T° test (°C) 35	+	+	+	+	+	+
42	+	-	+	-	+	-
Glucose	+	+	+	+	+	+
Fructose	+	+	+	+	+	+
Maltose	+/−	+/−	+/−	+/−	+/−	+/−
Mannitol	+/−	+/−	+/−	+/−	+/−	+/−
Arabinose	+/−	+/−	+/−	+/−	+/−	+/−
Raffinose	+/−	+/−	+/−	+/−	+/−	+/−
Trehalose	+/−	+/−	+/−	+/−	+/−	+/−
Sucrose	+/−	+/−	+/−	+/−	+/−	+/−
Lactose	+/−	+/−	+/−	+/−	+/−	+/−
Dulcitol	+/−	+/−	+/−	+/−	+/−	+/−

Table 2: Selective culture media and conditions for enumeration and isolation of lactic acid bacteria.

**LAB group**	**Media**	**T (°C)**	**Duration (h)**	**Incubation Condition**
Lactobacilli	MRS	30	48-72	Aerobic
Lactococci and Streptococci	M17	37	48-72	Aerobic
Lactobacillus	SL Rogassa	37	48-72	Aerobic
Bifidobacteria	MRS+cysteine (0.5%)	30	48-72	Anaerobic

Table 3: Colony characteristics and bacterial count of isolated Lactobacillus from milk and milk product samples

Type of sample (Sample No.)	Media	Colony characteristics
**Raw Milk (1)**	MRS	Yellow to white smooth creamy circular
	M17broth+agar powder	Pink to white circular
	Rogassa + acetic glacial acid (1.3ml/L)	Pinpoint spindle yellowish white
	MRS+cysteine (0.5%)	Small creamy white circular
**Raw milk (2)**	MRS	White pinpoint
	M17	Small whitish yellow
	Rogassa	Medium white small circular colony
	MRS+cysteine	Large flat colony
**Raw milk (3)**	MRS	spindle pinpoint
	M17	white shiny mucoid large
	Rogassa	Small spindle white
**Raw milk (4)**	MRS+cysteine	Small circular white
	MRS	White small pinpoint
	M17	Large buttery
**Raw milk (5)**	Rogassa	Small to medium white
	MRS+cysteine (0.05%)	Small white creamy
	MRS	Small white circular colony
	M17	Grayish white large circular
**Raw milk (6)**	Rogasa	Small yellowish colony
	MRS+cysteine	White flat circular
	MRS	a. white creamy large circular
		b. small white pinpoint(spindle)
	M17	a. small yellowish pinpoint
		b. large white circular
	Rogasa	a. small white creamy circular
		b. yellowish spindle pinpoint
	MRS+cysteine	a. small whitish circular
		b. large creamy circular spindle
		c. yellowish mucoid
	MRS	a. yellowish white circular

**Raw milk (7)**		b. small spindle pinpoint
	M17	a. large yellowish creamy white circular
		b. small yellowish spindle
	Rogasa	a. Large white circular colony
		b. small yellowish spindle
	MRS+cysteine	a. small creamy white circular
		b. small tiny pinpoint
	MRS	White small circular colony
**Yoghurt (1)**	M17	a. large white
		b. medium to small white
		c. small white
	Rogasa	a. large creamy white
		b. small white circular
	MRS+cysteine	a. large mucoid shiny
		b. small white circular
		c. irregular shape colony
**Yoghurt (2)**	MRS	a. white creamy circular
		b. small pinpoint
	M17	small white pinpoint
	MRS+cysteine	a. white creamy
		b. small grayish white
		a. white large
		b. small white
**Yoghurt (3)**	MRS	a.smallspindle pinpoint
		b.small whitish grey
	M17	small pin-point colony
	Rogasa	a.white creamy circular
		b.small whitish grey
	MRS+cysteine	a.large white circular colony
		b.small pinpoint spindle
**Yoghurt (4)**	MRS	a.large white creamy circular
		b.small whitish spindle
	M17	a.white large circular
		b.small pinpoint
	Rogasa	a.white creamy circular
		b.yellowish white spindle
	MRS+cysteine	a. white creamy large circular
		b. small yellowish spindle

**Yoghurt (5)**	MRS	a. whitish creamy circular
		b. small pinpoint spindle
	M17	a.large yellow creamy circular
		b.small whitish spindle
	Rogasa	a.large white circular
		b.yellowish spindle
	MRS+cysteine	a.large yellowish creamy white
		b.small white circular
**Cheese (1)**	MRS	a.white small circular colony
		b.spindle white grey
		c.yeast like colony
	M17	a.white creamy small
		b.small pinpoint
	Rogasa	a.medium white colony
		b.small pinpoint
	MRS+ cysteine	a.large white flat circular
		b.small white
**Cheese (2)**	MRS	a.large white circular
		b.small white circular
	M17	a.white cottony circular
		b.small white colony
	Rogasa	white circular
	MRS+ cysteine	a.white large circular
		b.small white
		a.white large shiny round
**Cheese(3)**	MRS	
		b.small white round
	M17	a.white large mucoid shiny round
		b.yellow fine spindle
	Rogasa	a.large white round
		b.small white spindle
	MRS+cysteine	a.small white milky
		b.large white spindle
**Cheese (4)**	MRS	a. large white round
		b. small white spindle
	M17	pinpoint grey spindle
	Rogasa	small yellow round
	MRS+cysteine	a. small grey Spindle

	b. large flat white

Table-4: Carbohydrates fermentation of lactic acid bacteria isolated from raw cowmilk, yoghurt and cheese in MRS media.

Sample. No.	Gram reaction	Catalase test	Glucose	Fructose	Maltose	Arabinose	Mannitol	Trehalose	Raffinose	Sucrose	Lactose	Dulcitol	BCP	Bacterial Morphology	Isolated presumptive genus
1	+	_	+	+	+	+	+	_	_	_	+	_	+	Rod	Lactobacilli
2	+	_	_	_	_	_	+	_	_	+	+	_	_	Rod	Lactobacilli
3	+	_	+	+	+	+	+	v	_	_	+	+	_	Rod	Lactobacilli
4	+	_	_	+	_	_	_	_	_	_	+	_	_	Cocci to coccobacilli	Leuconostoc
5	+	_	+	+	+	+	+	_	_	_	V	_	_	Cocci in chains	Pediococcus
6	+	_	+	+	V	+	+	+	_	_	_	_	+	Rod	Lactobacilli
7	+	_	+	+	_	+	+	+	_	_	+	_	_	Rod	Lactobacilli
8	+	_	+	+	+	+	+	+	_	+	_	_	_	Rod	Lactobacilli
9	+	_	+	+	_	_	+	+	_	+	_	_	+	Cocci in pairs	Lactococcus
10	+	_	+	+	_	_	+	+	_	+	_	_	_	Cocci	Lactococcus
11	+	_	+	+	+	+	+	v	+	_	+	_	+	Rod	Lactobacilli
12	+	_	+	+	V	+	_	+	_	+	_	_	+	Cocci	Lactococcus
13	+	_	+	+	+	+	+	+	v	+	+	_	_	Cocci	Pediococcus
14	+	_	+	+	+	_	_	+	_	+	+	_	+	Rod	Lactobacilli
15	+	_	+	+	+	_	_	+	_	+	+	_	+	Rod	Lactobacilli
16	+	_	+	_	_	_	_	_	_	+	+	_	_	Rod	Lactobacilli

BCP= Bromocresol purple dye,V=Variable,(+) = positive,(-) = negative

1,2,3,4,7,11,13 = milk sample, 5,8,9,12,14 = yoghurt, 6,10,15,16 = cheese

Table-5: Carbohydrates fermentation of lactic acid bacteria isolated from raw cow milk, yoghurt and cheese in M17 broth + agar powder

Sample. No.	Gram reaction	Catalase test	Glucose	Fructose	Maltose	Arabinose	Mannitol	Trehalose	Raffinose	Sucrose	Lactose	Dulcitol	BCP	Bacterial Morphology	Isolated presumptive genus
**1**	+	_	+	+	+	+	+	_	_	+	+	_	_	Cocci	Lactococcus
**2**	+	_	_	+	+	_	_	+	+	_	+	_	+	Rod	Lactobacilli
**3**	+	_	+	_	_	+	v	+	_	_	_	_	_	Cocci	Lactococcus
**4**	+	_	_	+	+	+	+	_	_	_	+	_	_	Rod	Lactobacilli
**5**	+	_	+	V	+	+	+	_	_	_	+	_	_	Cocci	Lactococcus
**6**	+	_	+	+	_	v	_	+	+	+	+	_	+	Rod	Lactobacilli
**7**	+	_	+	_	+	_	_	v	+	+	_	_	+	Rod	Lactobacilli
**8**	+	_	+	+	+	+	+	+	_	_	+	_	_	Cocci	Lactococcus
**9**	+	_	_	+	_	+	_	_	_	_	_	_	_	Cocci	Lactococcus
**10**	+	_	+	V	+	_	+	_	_	_	+	_	_	Cocci	Lactococcus
**11**	+	_	+	_	_	+	_	+	+	+	+	_	+	Rod	Lactobacilli
**12**	+	_	_	+	_	+	_	_	_	_	+	_	+	Rod	Lactobacilli
**13**	+	_	+	V	+	+	+	_	v	+	+	_	_	Cocci	Lactococcus
**14**	+	_	+	+	+	+	v	+	_	_	+	_	_	Rod	Lactobacilli
**15**	+	_	+	_	+	_	+	_	_	_	_	_	+	Cocci	Lactococcus
**16**	+	_	+	+	V	_	_	_	_	+	+	_	+	Cocci	Lactococcus

BCP= Bromocresol purple dye, V=Variable, (+) = positive, (-) = negative,

1,2,3,4,7,11,13 = milk sample, 5,8,9,12,14 = yoghurt, 6,10,15,16 = cheese

Table-6: Carbohydrates fermentation of lactic acid bacteria isolated from raw cow milk, yoghurt and cheese in Rogassa agar medium.

Sample. No.	Gram reaction	Catalase test	Glucose	Fructose	Maltose	Arabinose	Mannitol	Trehalose	Raffinose	Sucrose	Lactose	Dulcitol	BCP	Bacterial Morphology	Isolated presumptive genus
1	+	_	+	+	-	_	_	_	**V**	+	+	_	+	Rod	Lactobacilli
2	+	_	+	_	+	_	_	+	_	_	+	+	+	Cocci	Lactococcus
3	+	–	+	+	+	_	_	+	_	+	_	_	_	Cocci	Lactococcus
4	+	_	+	_	_	_	+	_	+	+	_	+	_	Rod	Lactobacilli
5	+	_	+	+	_	+	_	_	_	_	_	_	_	Rod	Lactobacilli
6	+	_	+	_	+	_	+	_	_	+	+	_	+	Rod	Lactobacilli
7	+	_	_	V	_	-	-	-	-	-	_	_	_	Cocci	Lactococcus
8	+	_	+	+	+	+	+	v	+	+	+	_	+	Rod	Lactobacilli
9	+	_	_	+	_	_	+	_	_	_	_	_	_	Rod	Lactobacilli
10	+	_	+	_	+	_	_	_	_	_	+	_	_	Rod	Lactobacilli
11	+	_	+	+	_	+	_	_	_	+	+	_	+	Rod	Lactobacilli
12	+	_	_	_	+	+	v	+	+	+	_	_	+	Cocci	Lactococcus
13	+	_	+	+	_	_	+	+	+	_	+	_	_	Rod	Lactobacilli
14	+	_	V	_	_	_	_	v	+	+	+	_		Cocci	Lactococcus
15	+	_	+	+	+	+	_	+	_	+	+	_	+	Cocci	Lactococcus
16	+	_	_	_	_	_	+	+	+	+	_	_	_	Rod	Lactobacilli

BCP= Bromocresol purple dye, V=Variable, (+) = positive, (-) = negative,

1,2,3,4,7,11,13 = milk sample, 5,8,9,12,14 = yoghurt, 6,10,15,16 = cheese

Table-7: Carbohydrates fermentation of lactic acid bacteria isolated from raw cow milk, yoghurt and cheese in MRS+cysteine (0.5%)

Sample. No.	Gram reaction	Catalase test	Glucose	Fructose	Maltose	Arabinose	Mannitol	Trehalose	Raffinose	Sucrose	Lactose	Dulcitol	BCP	Bacterial Morphology	Isolated presumptive genus
1	+	_	+	+	_	_	+	+	+	+	_	_	+	Filamentous rod	Lactobacilli
2	+	_	+	+	+	_	_	+	V	_	_	_	+	Pleomorphic Rod	Bifidobacteria
3	+	_	_	_	+	+	+	V	_	+	_	_	_	Rod	Lactobacilli
4	+	_	v	+	_	+	_	_	_	_	_	_	_	Rod	Lactobacilli
5	+	_	+	+	_	+	+	_	_	_	+	_	+	Rod	Bifidobacteria
6	+	_	+	+	+	+	_	+	_	+	+	_	_	Rod	Lactobacilli
7	+	_	+	_	+	v	_	_	+	+	v	_	+	Rod	Lactobacilli
8	+	_	_	_	+	_	_	_	_	+	_	_	_	Rod	Lactobacilli
9	+	_	+	V	+	_	_	+	_	_	+	_	+	Filamentous rod	Lactobacilli
10	+	_	+	+	+	_	_	+	_	v	+	_	+	Pleomorphic rod	Bifidobacteria
11	+	_	+	+	+	+	_	_	_	_	+	_	_	Pleomorphic rod	Bifidobacteria
12	+	_	+		V	+	_	+	_	+	+	_	_	Rod	Lactobacilli
13	+	_	+	+	+	+	+	_	+	+	+	_	+	Pleomorphic rod	Bifidobacteria
14	+	_	+	+	+	+	_	+	_	+	+	+	+	Rod	Lactobacilli
15	+	_	+	+	V	+	+	_	_	_	+	_	+	Cocci	Lactococcus
16	+	_	+	_	_	_	V	+	_	+	+	_	_	Cocci	Lactococcus

BCP= Bromocresol purple dye, V=Variable, (+) = positive, (-) = negative

1,2,3,4,7,11,13 = milk sample, 5,8,9,12,14 = yoghurt, 6,10,15,16 = cheese
